# Stable transfection of protein kinase C alpha cDNA in hormone-dependent breast cancer cell lines

**DOI:** 10.1054/bjoc.2000.1326

**Published:** 2000-08-17

**Authors:** D A Tonetti, M J Chisamore, W Grdina, H Schurz, V C Jordan

**Affiliations:** Robert H. Lurie Comprehensive Cancer Centre, Northwestern University Medical School, Chicago, Illinois, 60611–3008, USA

**Keywords:** breast cancer, protein kinase C, tamoxifen resistance, AP-1, hormone-independent

## Abstract

An inverse relationship between protein kinase C (PKC) activity and oestrogen receptor (ER) expression in human breast cell lines and tumours has been firmly established over the past 10 years. To determine whether specific alterations in PKC expression accompany hormone-independence, we examined the expression of PKC isozymes in the hormone-independent human breast cancer cell clones MCF-7 5C and T47D:C42 compared with their hormone-dependent counterparts, MCF-7 A4, MCF-7 WS8 and T47D:A18 respectively. Both hormone-independent cell clones exhibit elevated PKCα expression and increased basal AP-1 activity compared with the hormone-dependent cell clones. To determine whether PKCα overexpression is sufficient to mediate the hormone-independent phenotype, we stably transfected an expression plasmid containing PKCα cDNA to the T47D:A18 and MCF-7 A4 cell lines. This is the first report of PKCα transfection in T47D cells. In contrast to MCF-7 cells, T47D has the propensity to lose the ER and more readily forms tamoxifen-stimulated tumours in athymic mice. We find that in T47D:A18/PKCα clones, there is concomitant up-regulation of PKC βI and δ, whereas in the MCF-7 A4/PKCα transfectants PKC ɛ is up-regulated. In T47D:A18, but not in MCF-7 A4, PKCα stable transfection is accompanied by down-regulation of ER function whilst basal AP-1 activity is elevated. Our results suggest PKCα overexpression may play a role in growth signalling during the shift from hormone dependent to hormone-independent breast cancers. © 2000 Cancer Research Campaign


*British Journal of Cancer* (2000) **83**(6), 782–791
© 2000 Cancer Research Campaign
doi: 10.1054/ bjoc.2000.1326, available online at http://www.idealibrary.com on IDEAL®

### Stable transfection of protein kinase C alpha cDNA in hormone-dependent breast cancer cell lines

**DA Tonetti, MJ Chisamore, W Grdina, H Schurz and VC Jordan**

Robert H. Lurie Comprehensive Cancer Centre, Northwestern University Medical School, Chicago, Illinois 60611–3008, USA

**Summary** An inverse relationship between protein kinase C (PKC) activity and oestrogen receptor (ER) expression in human breast cell lines and tumours has been firmly established over the past 10 years. To determine whether specific alterations in PKC expression accompany hormone-independence, we examined the expression of PKC isozymes in the hormone-independent human breast cancer cell clones MCF-7 5C and T47D:C42 compared with their hormone-dependent counterparts, MCF-7 A4, MCF-7 WS8 and T47D:A18 respectively. Both hormone-independent cell clones exhibit elevated PKCα expression and increased basal AP-1 activity compared with the hormone-dependent cell clones. To determine whether PKCα overexpression is sufficient to mediate the hormone-independent phenotype, we stably transfected an expression plasmid containing PKCα cDNA to the T47D:A18 and MCF-7 A4 cell lines. This is the first report of PKCα transfection in T47D cells. In contrast to MCF-7 cells, T47D has the propensity to lose the ER and more readily forms tamoxifen-stimulated tumours in athymic mice. We find that in T47D:A18/PKCα clones, there is concomitant up-regulation of PKC βI and δ, whereas in the MCF-7 A4/PKCα transfectants PKC ε is up-regulated. In T47D:A18, but not in MCF-7 A4, PKCα stable transfection is accompanied by down-regulation of ER function whilst basal AP-1 activity is elevated. Our results suggest PKCα overexpression may play a role in growth signalling during the shift from hormone dependent to hormone-independent breast cancers. © 2000 Cancer Research Campaign

**Keywords:** breast cancer; protein kinase C; tamoxifen resistance; AP-1; hormone-independent

Tamoxifen is the most often prescribed hormonal therapy for the treatment of hormone-dependent, oestrogen receptor (ER)/progesterone receptor (PR) positive breast cancer. However, approximately half of all breast cancers do not respond to hormonal therapy and these hormone-independent tumours are more difficult to manage. Hormone-independent breast cancer is encountered in the following settings: 1. following long-term therapy with the antioestrogen tamoxifen, often termed acquired resistance, 2. initially tamoxifen non-responsive despite the tumour expressing ER, and 3. tumours classified as ER negative upon diagnosis. The latter two categories are referred to as *de novo* resistant, or hormone-independent. These three classifications of tumours represent the most aggressive forms of breast cancer and an effective treatment modality is not yet available. We and others have explored the basis of tamoxifen resistance by examining key steps along the ER-mediated signal transduction pathway (Encarnacion et al, 1993; Johnston et al, 1993; Osborne, 1993; Wolf et al, 1993; Karnik et al, 1994; Wolf and Jordan, 1994; Tonetti and Jordan, 1995). However, to date, examination of this pathway has yielded relatively few clues concerning the molecular mechanism of tamoxifen resistance. Identification of a suitable molecular target in these tumour types would facilitate the development of a logical therapeutic approach.

It is well documented that ER and protein kinase C (PKC) activity and abundance is inversely related in breast cancer cell lines (Borner et al, 1987) and PKC is elevated in malignant versus normal breast tissue (Borner et al, 1987; O'Brian et al, 1989; Gordge et al, 1996). Furthermore increased AP-1 (activator protein-1) activity has been reported to occur in hormone-independent breast cancer cell lines and tumours (Dumont et al, 1996; Johnston et al, 1999). PKC is a family of serine-threonine protein kinases which is now comprised of at least 11 isozymes, α, βI, βII, γ, δ, ε, ζ, η, θ, ι/λ and μ (Dekker and Parker, 1994), and is a known upstream activator of the AP-1 signal transduction pathway. These isozymes have been categorized into three groups based mainly on their co-factor requirements and sequence similarity, substrate specificity, and marked tissue and intracellular distribution (Nishizuka, 1992; Kazanietz et al, 1993). Activation of PKCs by the tumour promoter 12-*O*-tetradecanoylphorbol-13-acetate (TPA) results in the expression of the *c-fos* and *c-jun* family of nuclear protooncogenes, and increased transcriptional activity of genes containing a TPA-responsive element (TRE) (Angel and Karin, 1991). The AP-1 complex, comprised of either a Fos-Jun heterodimer or a Jun-Jun homodimer, binds to the TRE and induces gene transcription (Angel et al, 1987b; Lee et al, 1987). PKC isozymes differ in their ability to mediate transcriptional activation through the TRE (Hata et al, 1993; Reifel-Miller et al, 1996). TPA, which specifically binds to and activates PKC, causes down-regulation of the ER by post-transcriptional destabilization of the mRNA (Saceda et al, 1991) and inhibition of ER function (Martin et al, 1995). Furthermore, since ER and PKC expression is inversely related (Borner et al, 1987), it is possible that PKC could play a fundamental role in the slip from hormone-dependent to hormone-independent breast cancer.

Therefore, there is sufficient evidence in the literature indicating an involvement of ER and PKC in AP-1 activation. In this study we have examined PKC isozyme expression and AP-1 activation in two hormone-independent human breast cancer cell lines,

*Received 22 September 1999*
*Revised 27 April 2000*
*Accepted 1 May 2000*

*Correspondence to:* DA Tonetti

MCF-7 5C (Jiang et al, 1992) and T47D:C42 (Pink et al, 1996) compared with their respective hormone-dependent counterparts, MCF-7 A4(WS8) and T47D:A18. We find the hormone-independent cell lines overexpress PKCα and have elevated basal AP-1 activity. To determine whether PKCα overexpression is sufficient to create both the hormone-independent phenotype and induce elevated basal AP-1 activity, we stably transfected PKCα into the hormone-dependent T47D:A18 and MCF-7 A4 cell lines. Transfection of PKCα into MCF-7 cells was previously reported by two independent investigators (Ways et al, 1995; Manni et al, 1996). Ways et al report that overexpression of PKCα caused a reduction in ER expression, decreased oestrogen-dependent gene expression and a more aggressive neoplastic phenotype. In contrast, Manni et al. reported a less aggressive phenotype in MCF-7 cells as a result of PKCα overexpression. Since we have both hormone-dependent and -independent pairs of each cell type, we set out to determine the effect of PKCα overexpression in T47D cells compared to MCF-7. This question is especially relevant since T47D cells lose the ER more readily (Pink et al, 1996) and form tamoxifen-resistant tumours in athymic mice at an accelerated rate compared with MCF-7 cells (MacGregor-Schaffer et al, 2000). In consideration of the well-documented inverse relationship between ER and PKC, T47D cells provide a more appropriate cellular context in which to address the role of PKCα overexpression on the hormone-dependent phenotype. We find that in T47D:A18 cells overexpressing PKCα, there is concomitant up-regulation of PKC βI and δ, whereas in MCF-7 A4/PKCα transfectants PKCε is up-regulated. In T47D:A18, but not in MCF-7 A4, PKCα stable transfection is accompanied by down-regulation of ER function whilst basal AP-1 activity is elevated. PKCα stable transfection is not sufficient, however, to allow the antioestrogen tamoxifen to switch on the AP-1 pathway in either T47D:A18 or MCF-7:A4 cells. These results suggest that in the breast cancer cell lines T47D:A18 and MCF-7 A4, PKCα, and consequently βI, δ and ε overexpression, is not sufficient to trigger AP-1 activation, and is not likely to singly lead to tamoxifen-stimulated tumour growth. However, PKCα overexpression does lead to decreased ER function, and may play a role in *de novo* hormone-independent breast cancers.

### MATERIALS AND METHODS

### Cell lines and culture conditions

MCF-7 WS8 and T47D:A18 are hormone-dependent human breast cancer cell clones that have been previously described (Jiang et al, 1992; Pink et al, 1996). MCF-7 A4 is a hormone-dependent single cell clone of MCF-7 cells obtained from the American Type Culture Collection (ATCC, Rockville, MD). MCF-7 WS8, A4 and T47D:A18 were maintained in phenol red-containing RPMI-1640 medium supplemented with 10% FBS as previously described (Jiang et al, 1992; Pink et al, 1996). The hormone-independent MCF-7 5C cell clone was maintained in phenol red-free MEM supplemented with 5% 3 × dextran-coated charcoal (DCC)-treated calf serum (oestrogen-free media) as previously described (Jiang et al, 1992). The hormone-independent T47D:C42 clone was passaged in phenol red-free RPMI-1640 supplemented with 10% 3 × DCC-treated FBS (oestrogen-free media) as previously described (Pink et al, 1996). Prior to transient transfection experiments and RNA isolation, all cell lines were placed in oestrogen-free media for 3 days.

### Poly A$^+$ RNA isolation and Northern blot

Total RNA was isolated from cell lines using the Trizol reagent (GibcoBRL, Rockville, MD) prior to isolation of poly A$^+$ RNA using the PolyATract mRNA isolation kit (Promega, Madison, WI). Equal amounts of polyA$^+$ RNA were loaded per lane (4–5 μg), electrophoresed in a 1% agarose-0.66M formaldehyde gel, transferred to a Magnagraph nylon membrane (MSI, Westborough, MA) and the RNA was fixed to the membrane by UV cross-linking. The PKC α, δ, ε and η cDNA clones were obtained from ATCC (Rockville, MD) and the following cDNA fragments were labelled with [α-$^{32}$P]dCTP by random priming: a 1.3 Kb *Eco* RI PKC α fragment from phPKC-α7; a 1.7 Kb *Eco* RI PKC δ fragment from the I.M.A.G.E. Consortium Clone ID 236079; a 2.2 Kb *Nhe* I PKC ε fragment from pBluebac/PKC ε; a 1.7 *Pvu* II PKC fragment from pAcMP3/PKC eta. Equivalent loading of mRNA in each lane was verified by stripping and reprobing with β-actin cDNA.

### Partial PKC Purification and Western Blot

A PKC-enriched fraction was purified from at least 4 x $10^8$ cells by DEAE-cellulose anion exchange column chromatography as previously described (Tonetti et al., 1992). Protein concentration was measured using the Bio-Rad Dc protein assay system (Bio-Rad Laboratories, Hercules, CA). A SDS-8% polyacrylamide gel was loaded with equal amounts of protein (50-100 μg/lane) and pre-stained molecular weight standards to approximate the protein size. Following electrophoresis, protein was transferred to Hybond-ECL nitrocellulose membranes (Amersham Life Sciences, Buchinghamshire, England) by semi-dry electroblot transfer. PKC isozyme-specific monoclonal (PKCα, Transduction Laboratories, Lexington, KY) and polyclonal antibodies βI, βII, δ, ε, η, Santa Cruz Biotechnology, Santa Cruz, CA), were diluted as follows in TBS-T (20 mM Tris, pH 7.6; 137 mM NaCl; 0.1% Tween 20) containing 5% dry milk: α, 1:1000; βI, βII and δ, 1:500; ε, η, 1:250. The ECL Western blotting detection system (Amersham Life Science) was used to visualise the PKC bands. Equal loading of total protein per lane was assessed by Coomassie Brilliant Blue staining of a gel run in parallel or by stripping and reprobing with a β-actin monoclonal antibody (Sigma Chemical, St. Louis, MO).

### Generation of MCF-7 and T47D PKCα stable transfectants

The pSPKCα expression plasmid was generously provided by Dr Kirk Ways (Lilly Research Laboratories) (Reifel-Miller et al, 1996). MCF-7 A4 or T47D:A18 cells were suspended in serum-free medium (1 × $10^7$ cells/ml), mixed with 10 μg plasmid DNA and electroporated (250 V, 950 μF). The transfection mixture was added to 10 ml RPMI media (phenol red +) containing 10% FBS and seeded into 100 mm tissue culture dishes. Following incubation for 2 days, the medium was replaced with medium containing G418 (500 μg/ml media). The range of transfection efficiencies was approximately one G418-resistant transfectant/$10^4$-$10^5$ MCF-7 or T47D cells, and one-third all G418-resistant T47D and one fourth of all G418-resistant MCF-7 cells also overexpressed PKCα. Individual colonies were picked following 3 weeks of selection and were screened for PKCα expression by Western blot. Cells were transfected with the pSVHNX-neo plasmid as a control. The clones designated MCF7/neo, MCF7/α12, MCF7/α29 and T47D:A18/neo, T47D:A18/α5 and T47D:A18/α20 were chosen for further characterization.

*© 2000 Cancer Research Campaign*

### Proliferation assay

The cell lines T47D:A18/neo, T47D:A18/PKCα5 and T47D:A18/PKCα20 were seeded at 3 × $10^4$ cells/ml in either phenol red-containing RPMI media or phenol red-free RPMI (for determination of hormone-independent proliferation) supplemented with 500 μg/ml G418 into T25 tissue culture flasks. Cells were counted on days 2–10.

### Growth assays

Cells were grown in oestrogen-free media for 2 days prior to each experiment and were seeded into 24-well plates (15 000 cells/well) (Day 0). The following day (Day 1) medium containing either ethanol (control), E2 ($10^{-13}$–$10^{-7}$ M) or 4-OHT ($10^{-12}$–$10^{-6}$ M) was added. All compounds were dissolved in 100% ethanol and added to the medium at a 1:1000 dilution. Medium was changed on Days 3 and 5. DNA content was determined on Day 6 using a fluorocolorimeter (Labarca and Paigen, 1980).

### ER protein expression

Nuclear extracts were prepared from MCF-7A4 and T47D:A18 cell clones using a freeze-thaw method (Chen et al, 1996). ER expression was assessed by Western blot analysis using the ER mouse monoclonal antibody AER311 (human cross-reactive) (Neomarkers, Fremont, CA) followed by band detection with the ECL Western blotting kit (Amersham).

### Transient transfections and luciferase activity

A TRE-tk-Luc reporter plasmid was constructed by inserting a synthetic TRE derived from the human collagenase gene (5′ ATGAGTCAGA 3′) in tandem (4 copies) into the pT109luc vector (Nordeen, 1988). The ERE-tk-Luc plasmid contains three tandem copies of the vitellogenin ERE sequence inserted into pT109luc (Catherino and Jordan, 1995). Either the TRE-tk-luc or ERE-tk-luc plasmid was transiently co-transfected with the β-galactosidase (β-gal) expression plasmid pCMVβ (for the purpose of transfection efficiency normalization) into both the MCF-7 and the T47D:A18 stable clones by electroporation. Fifteen to 18 hours following transfection, the TRE-tk-Luc transfected cells were treated with either phorbol 12-myristate 13-acetate (PMA) ($10^{-7}$ M); 4-hydroxytamoxifen (4-OHT) ($10^{-7}$ M) or 17β-oestradiol (E2) ($10^{-9}$ M). The ERE-tk-Luc transfected cells were treated with E2 ($10^{-9}$ M), 4-OHT ($10^{-7}$ M), or ICI 182,780 ($10^{-7}$ M). Luciferase and β-gal activity was assayed 20 hours (ERE-tk-Luc transfection) or 5 and 20 hours (TRE-tk-Luc) following treatment using the Dual-Light Assay System (Tropix).

### Statistical analysis

When comparing to 1 as in Figures 4 and 9A, data were analysed using a one sample, one-tailed t-test. When comparing groups, data were analysed using one-way analysis of variance. Significant differences were indicated when $P < 0.05$.

### RESULTS

### PKC expression in hormone-dependent and hormone-independent breast cancer cell lines

Elevated PKC expression (upstream activator of AP-1) is reported to correlate with hormone-independent breast cancer (Wyss et al, 1987; O'Brian et al, 1989; Davidson and Kennedy, 1996; Gordge et al, 1996). We have examined two pairs of hormone-dependent and hormone-independent human breast cancer cell lines to determine whether a particular PKC isozyme(s) is associated with hormone independence. MCF-7 5C and T47D:C42 cell clones were derived from their respective parental cell lines MCF-7 and T47D by long-term culture in oestrogen-free media (Jiang et al, 1992; Pink et al, 1996) and are both E2 and antioestrogen (4-OHT and ICI 164,384) insensitive. The T47D:C42 cell clone has lost the expression of ERα, whereas the MCF7 5C cell clone exhibits reduced ERα expression (Figure 1) and impaired ER function (Jiang et al, 1992). We have compared PKC isozyme expression in these two hormone-independent breast cancer cell lines with their respective hormone-dependent counterpart cell clones, MCF-7

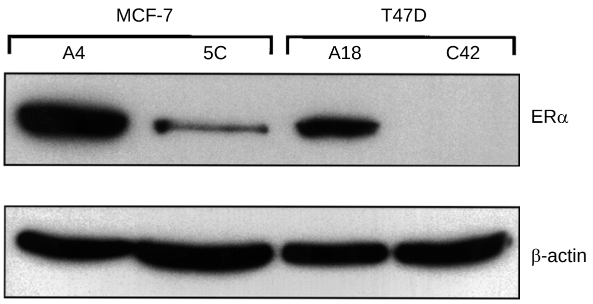

**Figure 1** ERα expression in hormone-dependent and hormone-independent breast cancer cell lines. Nuclear extracts were prepared from all breast cancer cell lines and a Western blot was performed using the ERα-specific monoclonal antibody, AER-311, that recognizes a 67 kDa protein. Equivalent protein loading was assessed by stripping the membrane and re-probing with a β-actin monoclonal antibody

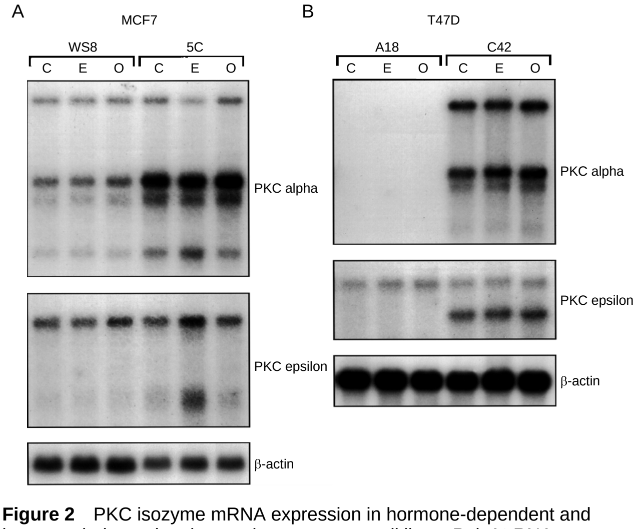

**Figure 2** PKC isozyme mRNA expression in hormone-dependent and hormone-independent human breast cancer cell lines. PolyA+ RNA was isolated (as described in 'Materials and Methods') following 24 hours treatment: C, control (ethanol vehicle); E, E2 $10^{-9}$ M; O, 4-OHT $10^{-7}$ M. A Northern blot was done by hybridization with PKCα, ε and β-actin cDNAs. (**A**) MCF-7 clones WS8 (hormone-dependent) and 5C (hormone-independent). (**B**) T47D clones A18 (hormone-dependent) and C42 (hormone-independent). Approximate sizes of specific messages were as follows: PKCα – 9.5, 3.7 and 3.2 kb; PKCε – 7.4 and 6.8. These results were reproduced at least three times and the Northern blot shown is a representative experiment

 *© 2000 Cancer Research Campaign*

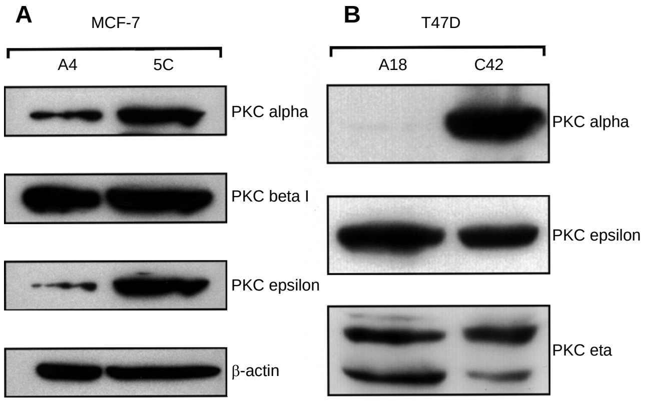

**Figure 3** PKCα protein expression in MCF-7 and T47D cell clones. A PKC-enriched fraction was isolated from the MCF-7 (**A**) and T47D (**B**) breast cancer cell lines and analysed by Western blot using specific antibodies to PKCα, βI, and ε isozymes, recognizing 82 kDa, 80 kDa and 90 kDa proteins, respectively. Equivalent protein loading was assessed by stripping the membrane and re-probing with a β-actin monoclonal antibody

A4(WS8) and T47D:A18, by both Northern and Western blot analysis.

PKC α, δ, ε, and η mRNAs were detected in all of the cell lines by Northern blot. The hormone-independent MCF-7 5C exhibits elevated expression of the PKCα transcripts compared with the hormone-dependent WS8 clone (Figure 2A). The MCF-7 WS8 and 5C clones express similar levels of PKC ε (Figure 2A) δ and η mRNAs (results not shown). Treatment of these cell clones with either E2 or 4-OHT did not alter the expression level of the PKCα isozyme transcripts examined, however there is a slight up-regulation of PKCε in response to E2 treatment (Figure 2A). Similarly, whereas the T47D:A18 (ER-positive, hormone-dependent) and C42 (ER-negative, hormone-independent) clones express comparable levels of PKC δ and η mRNAs in the untreated, E2 or 4-OHT-treated conditions (results not shown), PKC α and ε mRNAs are more abundant in the hormone-independent T47D C42 cell line (Figure 2B). In particular, expression of PKCα mRNA is undetectable in the T47D:A18 cells in contrast to the abundant expression observed in the T47D:C42 cell clone, whereas only a smaller PKC ε mRNA is more abundant in the C42 cells compared with A18. Therefore, whereas the hormone-dependent cell clones (MCF-7 WS8 and T47D A18) express very low or undetectable levels of PKC α and ε mRNAs, the hormone-independent MCF-7 5C and T47D C42 express comparatively more abundant PKC α and ε transcript expression.

To determine whether the abundance of PKC α and ε mRNA expression is reflected in the amount of protein produced, we examined PKC isozyme expression by Western blot analysis. The relative PKCα protein levels expressed in MCF-7 5C compared with MCF-7 A4 correspond to the PKCα transcript abundance observed; PKCα protein is expressed at higher levels in the hormone-independent MCF-7 5C cells (Figure 3A). PKC ε protein is more abundant in MCF-7 5C compared with the A4, whereas the PKC ε basal mRNA levels are comparable (Figure 2A). There is no difference in PKC βI expression.

Accordingly, the overexpression of PKCα mRNAs directly correlates with the PKCα protein levels expressed in T47D:A18 and C42; PKCα protein is more abundant in the hormone-independent C42 cells relative to the A18 cells (Figure 3B). There is no difference in PKC ε or η expression and PKC δ is barely detectable (results not shown) in the T47D clones.

### Induction of AP-1 transcriptional activation

Elevated AP-1 activity is associated with tamoxifen-resistant breast cancer (Astruc et al, 1995; Dumont et al, 1996; Smith et al, 1999). Since PKC is a known upstream activator of the AP-1 pathway, we compared the amount of basal and treatment-induced AP-1 activity in the hormone-dependent and hormone-independent breast cancer cell lines. Following transient co-transfection of the TRE-tk-luc and pCMVβ-gal plasmids, the MCF-7 A4, 5C and T47D:A18, C42 cell lines were either untreated (control, ethanol vehicle) or treated with PMA ($10^{-7}$ M), E2 ($10^{-9}$ M) or 4-OHT ($10^{-7}$ M) for 5 and 24 hours prior to determining luciferase and β-gal activities. Basal AP-1 activity was elevated in both of the hormone-independent cell clones MCF-7 5C and T47D:C42 compared with their hormone-dependent counterparts MCF-7 A4 and T47D:A18 (Figure 4A). The basal AP-1 activity of the MCF-7 5C cell clone was 7-fold higher than the MCF-7 A4 clone, although this did not achieve statistical significance at the $P < 0.05$ level. However, the basal AP-1 activity of T47D:C42 was elevated 2.6-fold compared with T47D:A18 ($P < 0.05$). Treatment of the cells with $10^{-7}$ M PMA (activator of PKC) for 5 hours caused an induction of AP-1 activity in all cells lines, verifying the functionality of the reporter construct (Figures 4B and 4C). PMA induced AP-1 activity to a greater extent in MCF-7 A4 cells compared with MCF-7 5C cells (16-fold vs 5-fold) (Figure 4B), most likely due to the fact that MCF-7 5C cells already exhibit high basal AP-1 activity. PMA-induced AP-1 activation was comparable (2-fold) in both T47D:A18 and C42 cell lines. Neither E2 nor 4-OHT treatment of any of the cell lines resulted in transcriptional activation of AP-1 at 5 h (Figures 4B and 4C) or 24 h (results not shown). We can conclude that the hormone-independent breast cancer cell lines (MCF-7 5C and T47D C42) have elevated basal AP-1 activity compared with their hormone-dependent counterparts (MCF-7 A4 and T47D A18). However this activity is not regulated by E2 or 4-OHT treatment.

### Stable transfection of PKCα in hormone-dependent T47D:A18 and MCF-7 A4 breast cancer cells

Since we have determined that both hormone-independent cell lines overexpress PKCα, we set out to determine the role of this isozyme in hormone-independent breast cancer. Therefore, we stably transfected the PKCα expression plasmid, pSPKCα, into MCF-7 A4 and T47D:A18 cells. After screening several clones for PKCα expression by Western blotting, we chose to further characterise two clones from each cell line, MCF-7 A4/PKCα12 and α29 and T47D:A18/PKCα5 and α20.

To determine whether overexpression of PKCα would affect the expression of other PKC isozymes, we performed Western blots using antibodies recognizing PKC βI, δ, and ε (Figure 5). We find that in MCF-7, overexpression of PKCα causes concomitant up-regulation of PKC ε. This pattern of PKC isozyme expression is similar to the MCF-7 5C cell line (Figure 3A). In the T47D:A18 cell line however, PKC βI and δ are up-regulated (Figure 5), whereas this PKC expression pattern is not evident in the T47D:C42 cell line. Cross-regulation between PKCα and β was previously reported in MCF-7 cells (Ways et al, 1995; Manni et al, 1996), however cross-regulation between PKCα and PKCs δ and ε has not been reported.

*© 2000 Cancer Research Campaign*

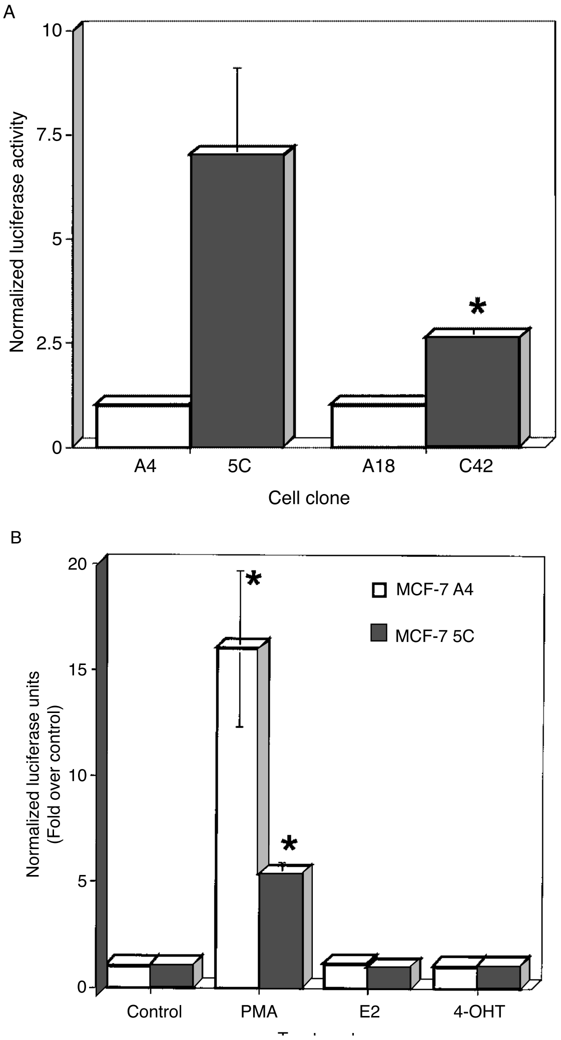

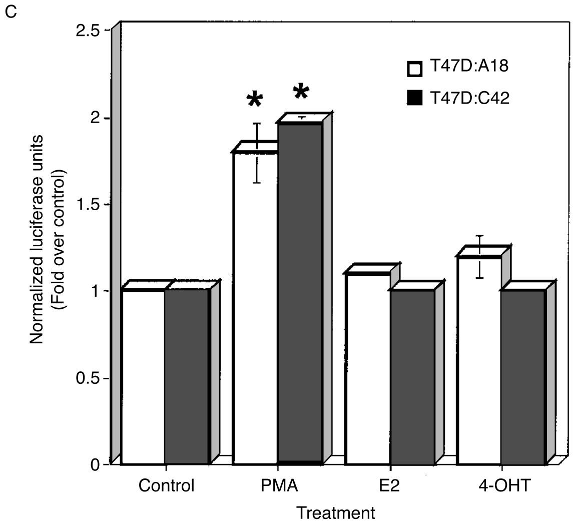

**Figure 4** Transcriptional activation of AP-1 in hormone-dependent and hormone-independent breast cancer cell lines. (**A**) Basal AP-1 transcriptional activity of MCF-7 A4, 5C and T47D:A18 and C42 cell clones. The TRE-tk-luc and pCMVβ plasmids were co-transfected into MCF-7 A4, 5C and T47D:A18, C42 breast cancer cell clones. Luciferase and β-gal activity was determined (as described in 'Material and Methods'). Normalized luciferase activity of the hormone-independent cell clones (MCF-7 5C and T47D A18) is expressed relative to their respective hormone-dependent cell clones (MCF-7 A4 and T47D C42, set = 1). (**B**) AP-1 transcriptional activation in response to PMA, E2 and 4-OHT in MCF-7 cell clones A4 and 5C, (**C**) T47D cell clones A18 and C42. Fifteen to 18 h following electroporation, the cells were treated with either ethanol vehicle (Control), PMA ($10^{-7}$ M), E2 ($10^{-9}$ M) or 4-OHT ($10^{-7}$ M). Luciferase and β-gal activities were measured 5 hours following treatment. The range of raw luciferase units obtained for all experiments for each cell line were as follows: MCF7 A4, 4703–15, 951; MCF-7 5C, 14, 103–125,588; T47D:A18, 800–2968; T47D:C42, 1114–2847. The background luciferase activity (transfection of pT109luc plasmid without AP-1 element) was between 150–200 raw luciferase units. All data is expressed as the normalized luciferase activity (mean ± SE) of 3 independent experiments performed in triplicate. A one sample, one-tailed t-test was used to determine significance compared to 1. *Indicates significance at $P < 0.05$

### The oestrogen-dependent phenotype of MCF-7 A4/PKCα and T47D:A18/PKCα stable transfectants

To examine whether the previously characterized inverse relationship of PKC and ER expression exists in our PKCα stable transfectants, we examined cell growth in response to E2, E2-induced transcriptional activation of an oestrogen response element (ERE), and ERα protein expression. Growth assays were performed measuring DNA content as the endpoint. We find that the T47D:A18/PKCα transfectants have a reduced oestrogen-induced growth response compared with the T47D:A18/neo control. Whereas the T47D:A18/neo cells demonstrated a 5-fold increase cell growth over the control, the PKCα stable clones exhibited only a 2-fold induction of growth following E2 treatment (Figure 6A). 4-OHT did not differentially affect the growth of T47D:A18/neo versus PKCα transfectants and was capable of competitively inhibiting the E2-induced growth response (results not shown). No difference in E2-(Figure 6B) nor 4-OHT-(results not shown) induced cell growth was apparent comparing the MCF-7 A4/neo, MCF-7A4/PKCα12 or α29 cell clones.

Transcriptional activation of an ERE was measured by transient transfection of an ERE-luciferase reporter plasmid into each cell line and luciferase activity was measured following 24 hour

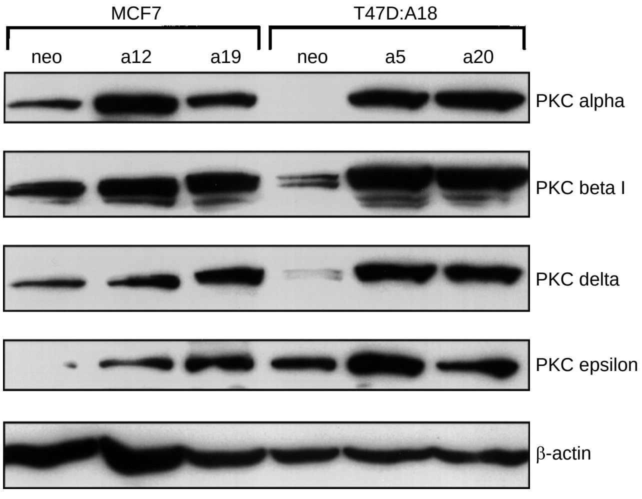

**Figure 5** Western blot analysis of PKC isozyme expression in T47D:A18 and MCF-7 A4 PKCα stable cell clones. PKC protein was partially purified from MCF-7 A4 and T47D:A18 neo and PKCα stable clones by DEAE-cellulose chromatography as described in Materials and Methods. Western blot analysis was performed using specific antibodies to PKCα, βI, δ, and ε isozymes recognizing 82, 80, 78 and 90 kDa proteins respectively. Equivalent protein loading was assessed by stripping the membrane and re-probing with a β-actin monoclonal antibody

© 2000 Cancer Research Campaign

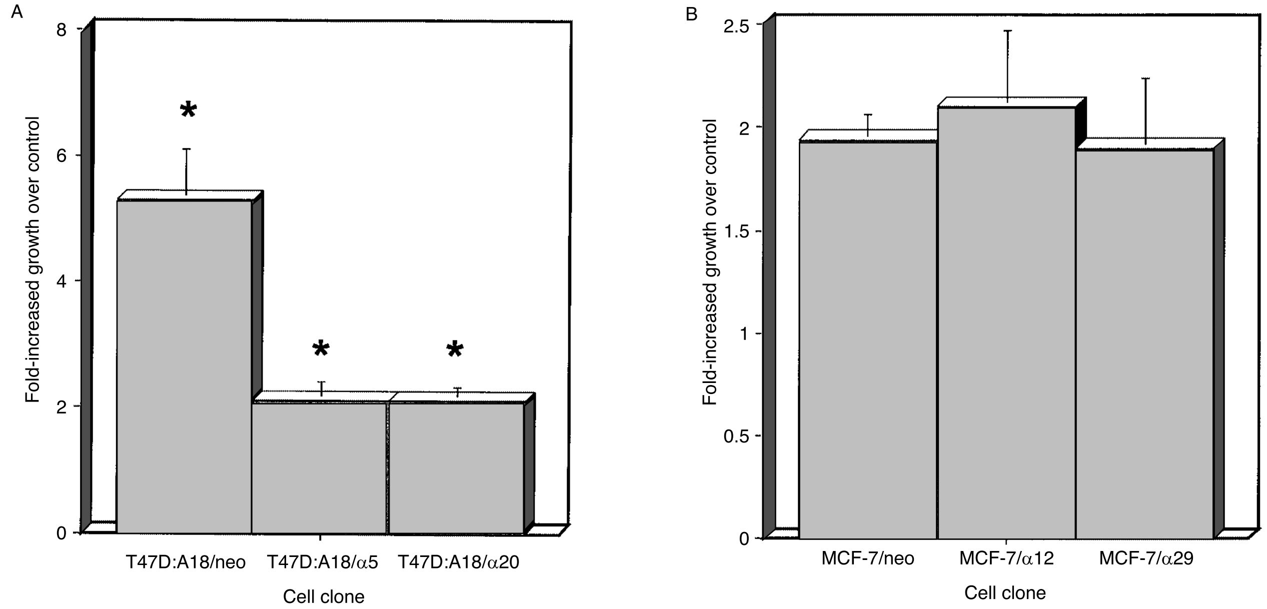

**Figure 6** E2-induced growth of T47D:A18 and MCF-7 A4 PKCα stable transfectants. Growth response curves to E2 was assessed in the PKCα and neo transfected clones of T47D:A18 by measuring DNA content 6 days following incubation in the presence of varying concentrations of E2 as described in Material and Methods. (**A**) Fold-increase in E2-induced cell growth (E2, $10^{-9}$ M) over control of T47D:A18/PKCα vs T47D:A18/neo transfectants. *Indicates groups are significantly different at *P* = 0.0039 using one-way ANOVA. (**B**) MCF-7 A4/PKCα vs MCF-7A4/neo transfectants (no differences among groups, ANOVA, *P* = 0.857). All data is expressed as the mean ± SE of 3 independent experiments performed in triplicate

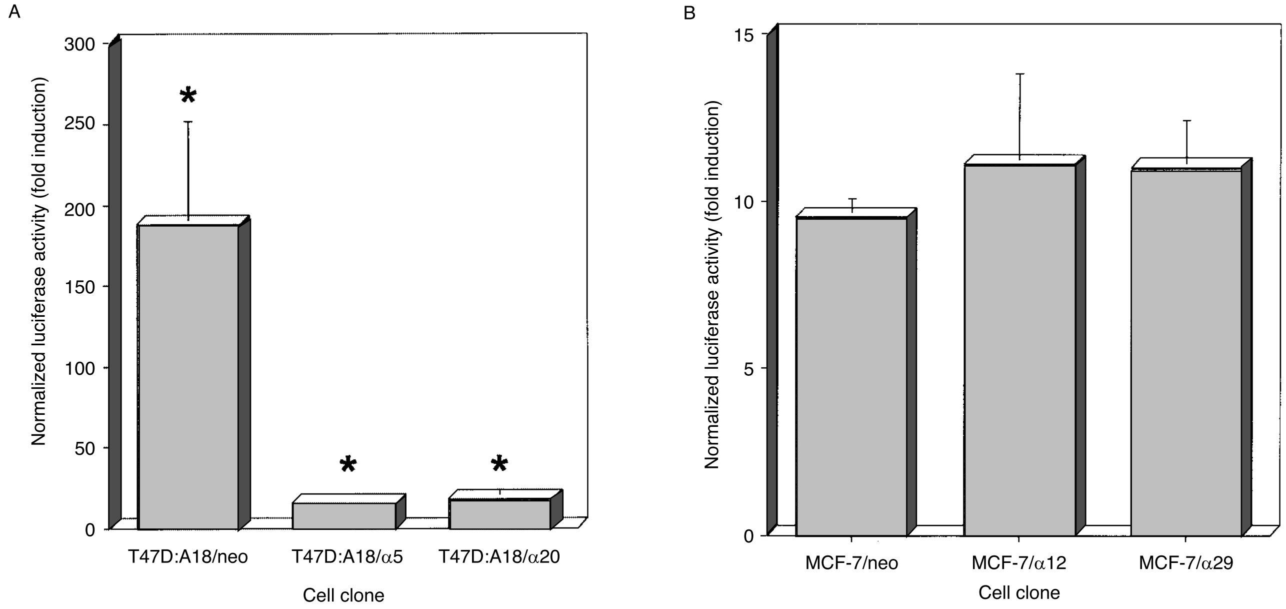

**Figure 7** ERE transcriptional activation in response to E2 in T47D:A18/PKCα and MCF-7/PKCα stable clones. ERE-TK-luc and pCMVβ plasmids were transiently co-transfected by electroporation into T47D:A18 (**A**) and MCF-7 (**B**) stable transfectants. Twenty-four hours following electroporation, the cells were treated with either ethanol vehicle (Control), E2 ($10^{-9}$ M) or ICI 182,780 ($10^{-7}$ M). Luciferase and β-gal activity was measured 20 hours following treatment. Data is expressed as the normalized E2-induced fold-increase over ICI 182,780 treatment (mean ± SE) of 3 independent experiments performed in triplicate. Data were analysed by one-way ANOVA. *Indicates significant difference among groups at *P* = 0.0263

treatment with E2. The ability of the T4D:A18/PKCα clones to activate transcription of an ERE-luciferase reporter plasmid was abrogated by an order of magnitude compared with the T47D:A18/neo clone (Figure 7A). Luciferase activity was induced 200-fold in the neo transfectants compared with only a 20-fold induction in the PKCα transfectants. The MCF-7 A4 clones however, displayed no difference in the ability to activate the ERE-tk-luc plasmid compared with the neo transfected control (Figure 7B). These results indicate an inverse relationship of PKCα and ER function in T47D:A18 cells as assessed by E2-induced growth response and activation of an ERE. The MCF-7 A4/PKCα clones, however, do not exhibit this inverse relationship

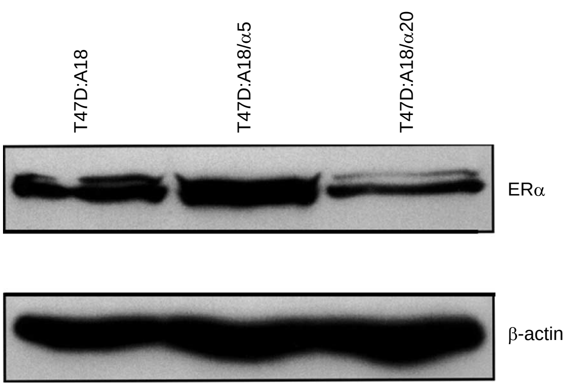

**Figure 8** ER expression in T47D:A18/PKCα stable clones. Nuclear extracts were prepared from each of the T47D:A18 stable clones as described in Materials and Methods. Equivalent amounts of nuclear extract protein (50 μg) were loaded per lane and a Western blot using an ER monoclonal antibody AER311 (Neomarkers) was performed recognizing a 67 kDa protein. Equal loading of protein per lane was assessed with a β-actin monoclonal antibody as was previously reported (Ways et al, 1995). Our results in MCF-7 cells may be due to either an insufficient level of PKCα to produce this effect, or may be attributed to differences in the particular MCF-7 cell clones used in the two laboratories. Alternatively, it may be the fact that PKCα overexpression in T47D:A18 leads to increased PKC βI and δ levels, whereas the MCF-7 A4/PKCα transfectants overexpress PKC ε.

To determine whether the impaired ER function in the T47D:A18/PKCα clones as exhibited by the reduced E2-induced growth response and transcriptional activation of an ERE was due to loss of ER expression, ERα protein levels were assessed by Western blotting. The T47D:A18 neo and PKCα5 clones appear to express comparable ERα, whereas the PKCα20 shows a slight decrease in ERα protein (Figure 8). This result suggests that although ERα protein is expressed, functionality is impaired by PKCα, and perhaps βI and δ over-expression. No difference in ERα levels were observed in the MCF-7 A4 transfectants (results not shown).

### Basal AP-1 activity in T47D:A18/PKCα stable transfectants

Since basal AP-1 activity is elevated in the hormone-independent T47D:C42 clone compared to T47D:A18, and T47D:C42 over-expresses PKCα, we wanted to determine whether the basal AP-1 activity was also elevated in the PKCα stable transfectants. We transiently transfected a TRE-luciferase reporter construct that contained four tandem repeats of the collagenase TRE sequence (Angel et al, 1987a). The PKCα clones exhibited 6–7-fold higher basal AP-1 activity compared with the neo transfectant (Figure 9A). These results suggest that overexpression of PKCα, and perhaps β and δ, is sufficient to raise the basal AP-1 activity to levels comparable to that of the hormone-independent T47D:C42 cell line. It is interesting to note that T47D:C42 cells do not exhibit PKC βI nor δ overexpression (results not shown). The MCF-7 A4 transfectants showed no increase in AP-1 basal activity (results not shown).

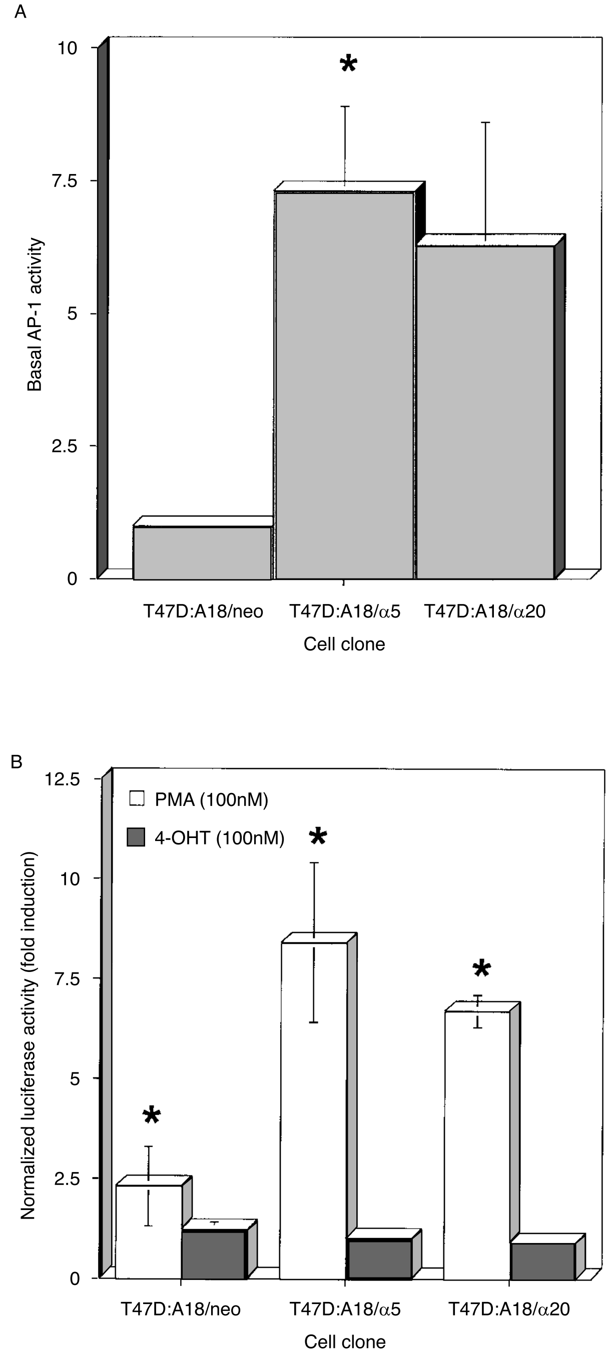

**Figure 9** AP-1 transcriptional activity of T47D:A18/PKCα stable clones. TRE-tk-luc and pCMVβ plasmids were transiently co-transfected by electroporation into T47D:A18/neo and PKCα stable transfectants. Luciferase and β-gal activity was measured 40 hours following transfection. (**A**) Basal AP-1 transcriptional activity shown as the normalized luciferase activity (luciferase/β-gal) of the PKCα clones expressed relative to the T47D:A18/neo clone (set = 1). *Indicates statistical significance at $P < 0.05$ using one-sample t-test. (**B**) AP-1 transcriptional activation in response to PMA and 4-OHT in T47D:A18/PKCα stable clones. Twenty-four hours following electroporation the cells were treated with either ethanol vehicle (control); PMA ($10^{-7}$ M) or 4-OHT ($10^{-7}$ M). Luciferase and β-gal activity was measured 20 hours following treatment. Data is expressed as the normalized luciferase activity (mean ± SE) of 3 independent experiments performed in triplicate. Data were analysed by one-way ANOVA. *Indicates statistical difference among groups treated with PMA at $P = 0.044$

*© 2000 Cancer Research Campaign*

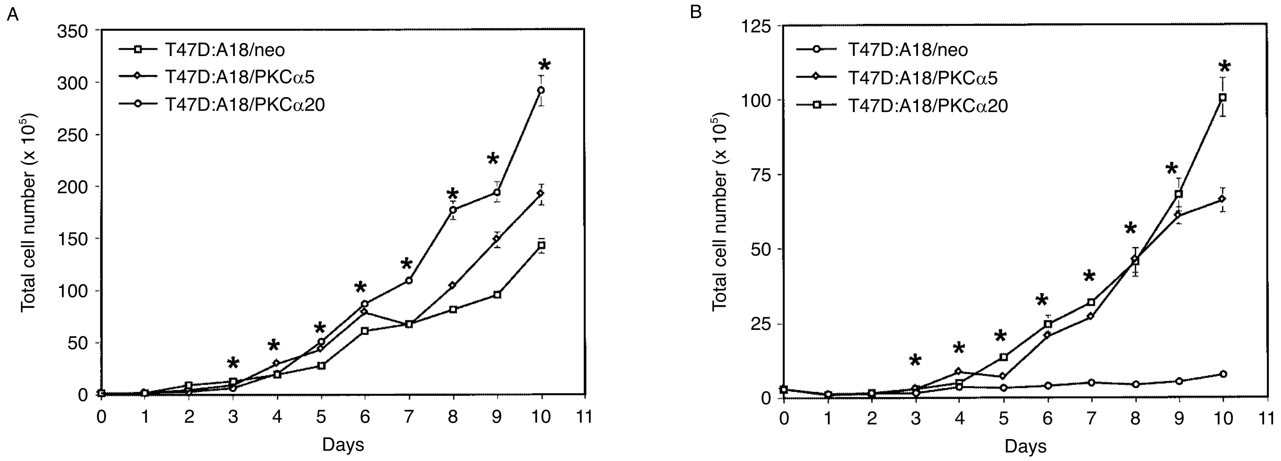

**Figure 10** Proliferation rate of T47D:A18/PKCα clones grown in medium containing whole serum (**A**) and E2-free medium (**B**). Growth rate was assessed by cell counting as described in Materials and Methods. The results are expressed as total cell number ± SE for each time point. These results are representative of three independent experiments. *Indicates statistical difference among groups at $P \leq 0.05$ (ANOVA)

### 4-OHT is incapable of activating AP-1 in T47D:A18/PKCα transfectants

We examined the ability of PMA, which is a potent PKC and AP-1 activator, to induce transcription of the TRE-tk-Luc reporter in the T47D:A18/PKCα transfectants. As we expected, the T47D:A18/PKCα transfectants exhibited a 6–8-fold higher AP-1 activation in response to PMA compared with the neo transfected T47D:A18 cells (Figure 9B). It was previously reported that tamoxifen is capable of activating AP-1 following long-term antioestrogen treatment of breast cancer cells in a PKC-dependent manner (Astruc et al, 1995). We hypothesized that PKC up-regulation may be a prerequisite step to tamoxifen-stimulated growth. Therefore we examined whether PKCα overexpression in T47D:A18 is sufficient to allow 4-OHT to activate AP-1. We found that 4-OHT did not activate AP-1 in these T47D:A18/PKCα transfectants (Figure 9B). This result is not surprising since the T47D:C42 (Pink et al, 1995) and T47D:A18/PKCα cell clones exhibit a hormone-independent phenotype and not a tamoxifen-stimulated phenotype.

### T47D:A18/PKCα proliferative rate

It was previously reported that overexpression of PKCα in MCF-7 cells leads to an increased proliferative rate compared to neo transfected MCF-7 cells (Ways et al, 1995). The T47D:A18/PKCα stable transfectants appear to have a similar growth rate as the T47D:A18/neo up until day 5. However after 5 days, T47D:A18/PKCα5 and α20 can grow to a higher cell density than the neo transfectant (Figure 10A). After 10 days, the T47D:A18/PKCα5 and α20 cell clones have not yet reached saturation density at $1.9 \times 10^7$ and $2.4 \times 10^7$ total cells respectively, and appear to be in good condition and remain attached to the plate. In contrast, the T47D:A18/neo cells at day 10 ($1.4 \times 10^7$ total cells) are beginning to detach from the plate and the remaining cells are in poor condition.

To determine whether PKCα overexpression is sufficient to confer hormone-independent growth we examined the rate of proliferation in oestrogen-free medium. We find that both T47D:A18/PKCα clones are able to grow at a statistically significant faster rate than the T47D:A18/neo clone in the absence of oestrogen between days 5–10 (Figure 10B).

### DISCUSSION

Approximately one half of all breast cancers are hormone-independent, and will not respond to hormonal therapy. Although tamoxifen is the endocrine treatment of choice for ER/PR positive breast cancers of all stages, eventually most tumours will become refractory to this therapy and the disease will recur. Based on reports in the literature, activation of the AP-1 signal transduction pathway may be involved in hormone-independent and tamoxifen-resistant breast cancers (Astruc et al, 1995; Webb et al, 1995; Dumont et al, 1996; Johnston et al, 1999; Smith et al, 1999). To explore this possible mechanism, we examined the PKC isozyme expression profile and AP-1 activity of MCF-7 and T47D breast cancer cell clone pairs that are hormone-dependent and -independent. We report a particular PKC isozyme, PKCα, is commonly overexpressed in both of the hormone-independent cell clones and is associated with elevated AP-1 activity. We established stable PKCα transfectants in the hormone-dependent T47D:A18 and MCF-7 A4 cell lines to determine whether PKCα overexpression is sufficient to confer elevated AP-1 activity and hormone-independence. We find that the T47D:A18/PKCα clones exhibited decreased ER function, increased hormone-independent growth rate, decreased contact inhibition and elevated basal AP-1 activity whereas the MCF-7/PKCα clones showed no effect on ER function or AP-1 activity.

The observation that ER and PKC expression are inversely related has been previously reported (Borner et al, 1987). In particular, inverse expression of PKCα and ER is reported in several breast cancer cell lines, and is associated with increased cell invasion (Morse-Gaudio et al, 1998; Platet et al, 1998; Johnson et al, 1999; Ng et al, 1999; Sun and Rotenberg, 1999). PKCα and ERα are inversely related in the hormone-independent cell lines T47D:C42 and MCF-7 5C. The T47D:C42 cells have lost the expression of ERα and express elevated PKCα protein, whereas the MCF-7 5C cells have reduced ERα expression, concomitant with elevated PKCα expression (Figures 1–3). ER function is

*© 2000 Cancer Research Campaign*

likely impaired in the 5C cells since oestrogen-stimulated PR expression and activation of an ERE-reporter construct is dramatically reduced in 5C cells (Jiang et al, 1992).

Other laboratories have previously reported stable transfection and overexpression of PKCα in the MCF-7 breast cancer cell line. Ways et al (1995) reported that MCF-7/PKCα stable transfectants exhibited reduced expression of the ER and of oestrogen-responsive genes, and resulted in a more aggressive neoplastic phenotype. Interestingly Manni et al (1996) reported that transfection of MCF-7 with PKCα produced no concomitant reduction in ER expression, and induced a less aggressive phenotype. These contrasting reports were attributed in part to differences in the expression of other PKC isozymes; Ways et al report upregulation of PKCβ, and downregulation of PKCs δ and η, whereas the transfectants described by Manni et al overexpressed only α and β isozymes (Manni et al, 1996). We find our MCF-7 5C cells overexpress PKCα, and unlike either study, PKC ε (Figure 3A). Similar to 5C cells, the MCF-7 A4/PKCα transfectants overexpress both PKCα and ε. Despite the fact that MCF-7 5C cells exhibit non-functional ER (reduced ERE activation, absence of E2-induced cell growth), the MCF-7/PKCα transfectants exhibited intact ER function. These results are in contrast to those of Ways et al (1995). Interestingly the T47D:A18/PKCα transfectants exhibited cross-up-regulation of PKC βI and δ, loss of ER function, increased AP-1 activity, hormone-independent growth and increased saturation density. This is the first report of PKCα transfection in T47D cells and is in agreement with the results in MCF-7 cells reported by Ways et al (1995). Our inability to observe these effects in MCF-7 may be due to either differences in MCF-7 clones, or perhaps insufficient levels of PKCα expression were achieved.

It has been reported that the AP-1 pathway may be activated by long-term tamoxifen treatment (Astruc et al, 1995) and tamoxifen-resistant breast cancer is associated with elevated AP-1 activity (Dumont et al, 1996; Johnston et al, 1999). Since PKCα is known to play a role in AP-1 signalling (Hata et al, 1993; Reifel-Miller et al, 1996), we investigated this pathway. It is important to point out, however, that the cell lines utilized in this study are hormone-independent. They were derived by long-term culture in oestrogen-free media and do not represent a model for long-term tamoxifen treatment. Although it was previously reported that both E2 and 4-OHT are capable of activating the AP-1 pathway (Astruc et al, 1995; Webb et al, 1995), E2 and 4-OHT did not induce AP-1 activity in either the MCF-7 nor T47D PKCα cell clones (Figure 4B and C). This is in contrast to the previous reports showing that E2 induces a 2-fold induction of an AP-1 reporter plasmid in MCF-7 cells (Philips et al, 1993; Webb et al, 1995). In addition, 4-OHT has been reported to activate AP-1 in a cell-type specific manner, it acts as an agonist in endometrial cell lines, but not in breast cell lines (Webb et al, 1995). In addition, prolonged treatment of MCF-7 cells with 4-OHT (4–12 days) was reported to activate an AP-1 reporter plasmid (Astruc et al, 1995). Clearly our hormone-independent cell clones have not been treated with 4-OHT long-term nor represent a model of tamoxifen-stimulated growth, and therefore such a response may not be expected.

Based on our results and others (Astruc et al, 1995; Webb et al, 1995; Dumont et al, 1996), activation of the AP-1 signal transduction pathway is a likely mechanism of hormone-independent breast cancer. We hypothesise that in the transition from tamoxifen-responsive to tamoxifen-resistant breast cancer, or in the case of *de novo* hormone-independent breast cancers, perhaps the equilibrium shifts from predominantly ER-mediated signalling to AP-1 signalling. The report of Webb et al (1995) provided evidence that the antioestrogen/ER complex can interact with the AP-1 components through a protein:protein interaction. However, in those cases of hormone-independence due to loss of ER or a non-functional ER, PKCα up-regulation may be an alternate route to AP-1 activation. At present the mechanism of PKCα up-regulation as well as the specific intermediate steps leading towards AP-1 activation are unknown. We suggest that perhaps the overexpression of PKCα may be a prerequisite step in initiating this switch in signalling. To directly test this hypothesis, we are using antisense technology to downregulate PKCα expression in tamoxifen-stimulated breast tumours in athymic mice in an attempt to abrogate tamoxifen-stimulated tumour growth. We have preliminary evidence that the T47D:A18/PKCα clones form tumours in ovariectomised athymic mice both in response to tamoxifen and in the absence of estrogen supplementation (Tonetti, unpublished observations). We are in the process of characterising these tumours. Identification of a specific PKC isozyme as a potential therapeutic target would be an important advance in the management of hormone-independent breast cancer.

### ACKNOWLEDGEMENTS

This work was supported in part by ACS, Illinois Division Grant #97–27 (D.A.T.), NIH RO1 CA79847–02 (D.A.T.) and the Lynn Sage Breast Cancer Foundation of Northwestern Memorial Hospital. We also wish to gratefully acknowledge the support of the Avon Breast Cancer Crusade. The generous gift of the pSPKCα expression plasmid was provided by Dr D Kirk Ways (Lilly Research Laboratories, Indianapolis, IN). The authors would like to thank Dr Alfred Rademaker for his advice on statistical analysis and the excellent technical assistance of Ms Shashikala Tanjore.

### REFERENCES

Angel P and Karin M (1991) The role of Jun, Fos and the AP-1 complex in cell-proliferation and transformation. *Biochim Biophys Acta* **1072**: 129–157

Angel P, Baumann I, Stein B, Delius H, Rahmsdorf HJ and Herrlich P (1987a) 12-O-tetradecanoyl-phorbol-13-acetate induction of the human collagenase gene is mediated by an inducible enhancer element located in the 5′-flanking region. *Mol Cell Biol* **7**: 2256–2266

Angel P, Imagawa M, Chiu R, Stein B, Imbra RJ, Rahmsdorf HJ, Jonat C, Herrlich P and Karin M (1987b) Phorbol ester-inducible genes contain a common cis element recognized by a TPA-modulated trans-acting factor. *Cell* **49**: 729–739

Astruc ME, Chabret C, Bali P, Gagne D and Pons M (1995) Prolonged treatment of breast cancer cells with antiestrogens increases the activating protein-1-mediated response: involvement of the estrogen receptor. *Endocrinology* **136**: 824–832

Borner CR, Wyss R, Regazzi RUE and Fabbro D (1987) Immunological quantitation of phospholipid/$Ca^{2+}$-dependent protein kinase of human mammary carcinoma cells: inverse relationships to estrogen receptors. *Int J Cancer* **40**: 344–348

Catherino WH and Jordan VC (1995) Increasing the number of tandem estrogen response elements increases the estrogenic activity of a tamoxifen analogue. *Cancer Lett* **92**: 39–47

Chen TK, Smith LM, Gebhardt DK, Birrer MJ and Brown PH (1996) Activation and inhibition of the AP-1 complex in human breast cancer cells. *Mol Carcinog* **15**: 215–226

Davidson N and Kennedy M (1996) Protein kinase C and breast cancer. In: R, D). and M, L. (eds) *Mammary tumor cell cycle, differentiation and metastasis* pp. 91–105. Kluwer Academic Pubishers.

Dekker LV and Parker PJ (1994) Protein kinase C–a question of specificity. *Trends Biochem Sci* **19**: 73–77

 © 2000 Cancer Research Campaign

Dumont JA, Bitoni AJ, Wallace CD, Baumann RJ, Cashman EA and Cross-Doersen DE (1996) Progression of MCF-7 breast cancer cells to antiestrogen-resistant phenotype is accompanied by elevated levels of AP-1 DNA-binding activity. *Cell Growth Differ* **7**: 351–359

Encarnacion CA, Ciocca DR, McGuire WL, Clark GM, Fuqua SA and Osborne CK (1993) Measurement of steroid hormone receptors in breast cancer patients on tamoxifen. *Breast Cancer Res Treat* **26**: 237–246

Gordge PC, Hulme MJ, Clegg RA and Miller WR (1996) Elevation of protein kinase A and protein kinase C activities in malignant as compared with normal human breast tissue. *Eur J Cancer* **32A**: 2120–2216

Hata A, Akita Y, Suzuki K and Ohno S (1993) Functional divergence of protein kinase C (PKC) family members. PKC gamma differs from PKC alpha and -beta II and nPKC epsilon in its competence to mediate-12-O-tetradecanoyl phorbol 13-acetate (TPA)- responsive transcriptional activation through a TPA-response element. *J Biol Chem* **268**: 9122–9129

Jiang SY, Wolf DM, Yingling JM, Chang C and Jordan VC (1992) An estrogen receptor positive MCF-7 clone that is resistant to antiestrogens and estradiol. *Mol Cell Endocrinol* **90**: 77–86

Johnson MD, Torri JA, Lippman ME and Dickson RB (1999) Regulation of motility and protease expression in PKC-mediated induction of MCF-7 breast cancer cell invasiveness [In Process Citation]. *Exp Cell Res* **247**: 105–113

Johnston SR, Haynes BP, Sacks NP, McKinna JA, Griggs LJ, Jarman M, Baum M, Smith IE and Dowsett M (1993) Effect of oestrogen receptor status and time on the intra-tumoural accumulation of tamoxifen and N-desmethyltamoxifen following short-term therapy in human primary breast cancer. *Breast Cancer Res Treat* **28**: 241–250

Johnston SR, Lu B, Scott GK, Kushner PJ, Smith IE, Dowsett M and Benz CC (1999) Increased activator protein-1 DNA binding and c-Jun NH2-terminal kinase activity in human breast tumors with acquired tamoxifen resistance. *Clin Cancer Res* **5**: 251–256

Karnik PS, Kulkarni S, Liu XP, Budd GT and Bukowski RM (1994) Estrogen receptor mutations in tamoxifen-resistant breast cancer. *Cancer Res* **54**: 349–353

Kazanietz MG, Areces LB, Bahador A, Mischak H, Goodnight J, Mushinski JF and Blumberg PM (1993) Characterization of ligand and substrate specificity for the calcium-dependent and calcium-independent protein kinase C isozymes. *Mol Pharmacol* **44**: 298–307

Labarca C and Paigen K (1980) A simple, rapid, and sensitive DNA assay procedure. *Anal Biochem* **102**: 344–352

Lee W, Mitchell P and Tjian R (1987) Purified transcription factor AP-1 interacts with TPA-inducible enhancer elements. *Cell* **49**: 741–752

MacGregor Schafer J, Lee ES, O'Regan RM, Yao K and Jordan VC (2000) Rapid development of tamoxifen-stimulated mutant p53 breast tumors (T47D) in athymic in mice. *Clin Cancer Res* ( In press)

Manni A, Buckwalter E, Etindi R, Kunselman S, Rossini A, Mauger D, Dabbs D and Demers L (1996a) Induction of a less aggressive breast cancer phenotype by protein kinase c-α and -β overexpression. *Cell Growth & Differentiation* **7**: 1187–1198

Martin MB, Garcia-Morales P, Stoica A, Solomon HB, Pierce M, Katz D, Zhang S, Danielsen M and Saceda M (1995) Effects of 12-O-tetradecanoylphorbol-13-acetate on estrogen receptor activity in MCF-7 cells. *J Biol Chem* **270**: 25244–25251

Morse-Gaudio M, Connolly JM and Rose DP (1998) Protein kinase C and its isoforms in human breast cancer cells: relationship to the invasive phenotype. *Int J Oncol* **12**: 1349–1354

Ng T, Shima D, Squire A, Bastiaens PI, Gschmeissner S, Humphries MJ and Parker PJ (1999) PKCalpha regulates betal integrin-dependent cell motility through association and control of integrin traffic. *Embo J* **18**: 3909–3923

Nishizuka Y (1992) Intracellular signaling by hydrolysis of phospholipids and activation of protein kinase C. *Science* **258**: 607–614

Nordeen SK (1988) Luciferase reporter gene vectors for analysis of promoters and enhancers. *Biotechniques* **6**: 454–8

O'Brian C, Vogel VG, Singletary SE and Ward NE (1989) Elevated protein kinase C expression in human breast tumor biopsies relative to normal breast tissue. *Cancer Res* **49**: 3215–3217

Osborne CK (1993) Mechanisms for tamoxifen resistance in breast cancer: possible role of tamoxifen metabolism. *J Steroid Biochem Mol Biol* **47**: 83–89

Philips A, Chalbos D and Rochefort H (1993) Estradiol increases and anti-estrogens antagonize the growth factor-induced activator protein-1 activity in MCF7 breast cancer cells without affecting c-fos and c-jun synthesis [published erratum appears in J Biol Chem 1993 Dec 5;268(34):26032]. *J Biol Chem* **268**: 14103–14108

Pink JJ, Jiang SY, Fritsch M and Jordan VC (1995) An estrogen-independent MCF-7 breast cancer cell line which contains a novel 80-kilodalton estrogen receptor-related protein. *Cancer Res* **55**: 2583–2590

Pink JJ, Bilimoria MM, Assikis J and Jordan VC (1996) Irreversible loss of the oestrogen receptor in T47D breast cancer cells following prolonged oestrogen deprivation [published erratum appears in Br J Cancer 1997;75(10):1557]. *Br J Cancer* **74**: 1227–1236

Platet N, Prevostel C, Derocq D, Joubert D, Rochefort H and Garcia M (1998) Breast cancer cell invasiveness: correlation with protein kinase C activity and differential regulation by phorbol ester in estrogen receptor-positive and -negative cells. *Int J Cancer* **75**: 750–756

Reifel-Miller AE, Conarty DM, KM, V, PW, I, DJ B and KA B (1996) Protein kinase C isozymes differentially regulate promoters containing PEA-3/12-O-tetradecanoylphorbol-13-acetate response element motifs. *J Biol Chem* **271**: 21666–21671

Saceda M, Knabbe C, Dickson RB, Lippman ME, Bronzert D, Lindsey RK, Gottardis MM and Martin MB (1991) Post-transcriptional destabilization of estrogen receptor mRNA in MCF-7 cells by 12-O-tetradecanoylphorbol-13-acetate. *J Biol Chem* **266**: 17809–17814

Smith LM, Wise SC, Hendricks DT, Sabichi AL, Bos T, Reddy P, Brown PH and Birrer MJ (1999) cJun overexpression in MCF-7 breast cancer cells produces a tumorigenic, invasive and hormone resistant phenotype. *Oncogene* **18**: 6063–6070

Sun XG and Rotenberg SA (1999) Overexpression of protein kinase Calpha in MCF-10A human breast cells engenders dramatic alterations in morphology, proliferation, and motility [In Process Citation]. *Cell Growth Differ* **10**: 343–352

Tonetti DA and Jordan VC (1995) Possible mechanisms in the emergence of tamoxifen-resistant breast cancer. *Anticancer Drugs* **6**: 498–507

Tonetti DA, Horio M, Collart FR and Huberman E (1992) Protein kinase C beta gene expression is associated with susceptibility of human promyelocytic leukemia cells to phorbol ester-induced differentiation. *Cell Growth Differ* **3**: 739–745

Ways DK, Kukoly CA, deVente J, Hooker JL, Bryant WO, Posekany KJ, Fletcher DJ, Cook PP and Parker PJ (1995) MCF-7 breast cancer cells transfected with protein kinase C-alpha exhibit altered expression of other protein kinase C isoforms and display a more aggressive neoplastic phenotype. *J Clin Invest* **95**: 1906–1915

Webb P, Lopez GN, Uht RM and Kushner PJ (1995) Tamoxifen activation of the estrogen receptor/AP-1 pathway: potential origin for the cell-specific estrogen-like effects of antiestrogens. *Mol Endocrinol* **9**: 443–456

Wolf DM and Jordan VC (1994) The estrogen receptor from a tamoxifen stimulated MCF-7 tumor variant contains a point mutation in the ligand binding domain. *Breast Cancer Res Treat* **31**: 129–138

Wolf DM, Langan-Fahey SM, Parker CJ, McCague R and Jordan VC (1993) Investigation of the mechanism of tamoxifen-stimulated breast tumor growth with nonisomerizable analogues of tamoxifen and metabolites. *J Natl Cancer Inst* **85**: 806–812

Wyss R, Fabbro D, Regazzi R, Borner C, Takahashi A and Eppenberger U (1987) Phorbol ester and epidermal growth factor receptors in human breast cancer. *Anticancer Res* **7**: 721–727

*© 2000 Cancer Research Campaign*
